# In Vitro Effect of 9,9′-Norharmane Dimer against Herpes Simplex Viruses

**DOI:** 10.3390/ijms25094966

**Published:** 2024-05-02

**Authors:** María Micaela Gonzalez, Maria Guadalupe Vizoso-Pinto, Rosa Erra-Balsells, Thomas Gensch, Franco M. Cabrerizo

**Affiliations:** 1 Instituto Tecnológico de Chascomús (CONICET-UNSAM), Av. Intendente Marino Km 8.2, CC 164 (B7130IWA), Chascomús 7130, Argentina; mgonzalez@intech.gov.ar; 2 Escuela de Bio y Nanotecnologías (UNSAM), San Martín 1650, Argentina; 3 Max von Pettenkofer Institute, Virology, National Reference Center for Retroviruses, Faculty of Medicine, LMU, D-80336 Munich, Germany; mgvizoso@fm.unt.edu.ar; 4 Instituto Superior de Investigaciones Biológicas (INSIBIO), CONICET-UNT, San Miguel de Tucumán 4000, Argentina; 5 Laboratorio Central de Cs. Básicas, Facultad de Medicina, Universidad Nacional de Tucumán, Tucumán 4000, Argentina; 6 Departamento de Química Orgánica, Facultad de Ciencias Exactas y Naturales, Universidad de Buenos Aires, Pabellón II, 3er P., Ciudad Universitaria, Buenos Aires 1428, Argentina; erra@qo.fcen.uba.ar; 7 Centro de Investigación en Hidratos de Carbono (CIHIDECAR), CONICET, Facultad de Ciencias Exactas y Naturales, Universidad de Buenos Aires, Naturales Pabellón II, 3er P. Ciudad Universitaria, Buenos Aires 1428, Argentina; 8 Institute of Biological Information Processing 1 (IBI-1; Molecular and Cellular Physiology), Forschungszentrum Jülich, Wilhelm-Jonen-Straße, 52428 Jülich, Germany

**Keywords:** β-carbolines, alkaloids, herpes simplex-1, herpes simplex-2, cellular uptake, cytotoxicity

## Abstract

Herpes simplex virus (HSV) infections are highly widespread among humans, producing symptoms ranging from ulcerative lesions to severe diseases such as blindness and life-threatening encephalitis. At present, there are no vaccines available, and some existing antiviral treatments can be ineffective or lead to adverse effects. As a result, there is a need for new anti-HSV drugs. In this report, the in vitro anti-HSV effect of 9,9′-norharmane dimer (nHo-dimer), which belongs to the β-carboline (βC) alkaloid family, was evaluated. The dimer exhibited no virucidal properties and did not impede either the attachment or penetration steps of viral particles. The antiviral effect was only exerted under the constant presence of the dimer in the incubation media, and the mechanism of action was found to involve later events of virus infection. Analysis of fluorescence lifetime imaging data showed that the nHo-dimer internalized well into the cells when present in the extracellular incubation medium, with a preferential accumulation into perinuclear organelles including mitochondria. After washing the host cells with fresh medium free of nHo-dimer, the signal decreased, suggesting the partial release of the compound from the cells. This agrees with the observation that the antiviral effect is solely manifested when the alkaloid is consistently present in the incubation media.

## 1. Introduction

Herpes simplex viruses type-1 (HSV-1) and type-2 (HSV-2) are highly prevalent human dsDNA viruses that belong to the *Alphaherpesvirinae* subfamily [1]. According to the latest epidemiological estimates (2016), 66.6% and 13.2% of the world’s population under 50 years old are infected with HSV-1 and HSV-2, respectively [2]. These viruses enter the body through mucous membranes or skin lesions, then migrate via axonal transport towards sensory nerve ganglia, where they establish latency and may reactivate several times throughout an individual’s lifespan [3].

Although these viruses generally cause mild diseases such as skin and mucosal injuries in genital and orofacial areas, they can occasionally produce severe diseases such as corneal blindness and encephalitis, which have a very high fatality rate (>70%) if left untreated [4]. Moreover, HSV-2 infection increases the risk of human immunodeficiency virus (HIV) acquisition and transmission in the general population and contributes to the HIV epidemic [5].

Acyclovir (ACV) and other nucleoside and nucleotide analogs have been the gold standard for HSV therapy and prophylaxis since the 1980s. Immunocompetent individuals generally require short-term treatments and show a low prevalence of ACV resistance (<1% of cases). In contrast, 3.5–10% of immunocompromised patients who need long-term therapies may develop HSV variants that are resistant to ACV [6]. In this case, second-line antivirals such as cidofovir and foscarnet are available. However, the former has not been approved for the treatment of herpes simplex viruses in humans, and both drugs have numerous side effects. Resistance cases have also been reported [1]. Therefore, the development of new antivirals based on different principles or mechanisms of action might contribute to improving the efficacy of current treatments, particularly against ACV-resistant HSV viruses.

β-carbolines (βCs) are a group of alkaloids that occur naturally and have remarkable pharmacological properties [7]. Currently, there are nine βCs available on the pharmaceutical market, which have demonstrated antihypertensive, vasodilator and antipsychotic effects [8]. Moreover, both natural and synthetic βCs have exhibited various effective in vitro actions against cancerous cells [9,10], parasites [8,11,12], fungi [13], bacteria [14] and viruses [15]. Some studies have also demonstrated the efficacy of βCs in vivo [9,16]. Their spectrum of antiviral activity includes DNA and RNA viruses, such as HSV [15,16,17], HIV [18], poliovirus (PV) [19], tobacco mosaic virus (TMV) [20], dengue virus 2 (DENV-2) [21] and avian and human influenza A viruses [22]. Particularly, HSV-1 was the first virus reported to be affected by certain βC alkaloids (Eudistomins) obtained from the colonial tunicate *Eudistoma olivaceum* [23]. Since then, various studies have proven the anti-HSV activities of βC-monomers including harmine [17], harmaline [16], 9-methyl-norharmane, 9-methyl-harmane and 6-methoxyharmane [15]. Interestingly, the latter study demonstrated that N(9)-methyl substitution enhances the anti-HSV effect. Moreover, unusual heterodimeric structures containing a rare tetracyclic moiety connected to a classic tricyclic one by a C15-C1’ bridge isolated from *Peganum harmala* seeds have also been shown to be active against HSV-2 [24]. However, the antiviral activity of βC dimers has not been studied extensively.

**Figure 1:**
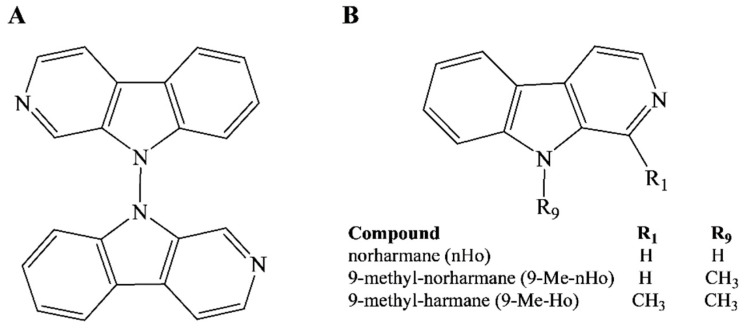
Chemical structures of (**A**) 9,9′-norharmane dimer and (**B**) three related monomers.

In this study, we explore the antiherpetic activity and subcellular localization of a symmetric 9,9′-norharmane dimer (nHo-dimer, Figure 1A) and compare the results with those obtained for two related monomers, norharmane (nHo) and 9-methyl-norharmane (9-Me-nHo), as well as 9-methyl-harmane (9-Me-Ho) (Figure 1B) and ACV, which have been previously reported in the literature.

## 2. Results

### 2.1. Cytotoxic and Antiproliferative Effect

**Figure 2:**
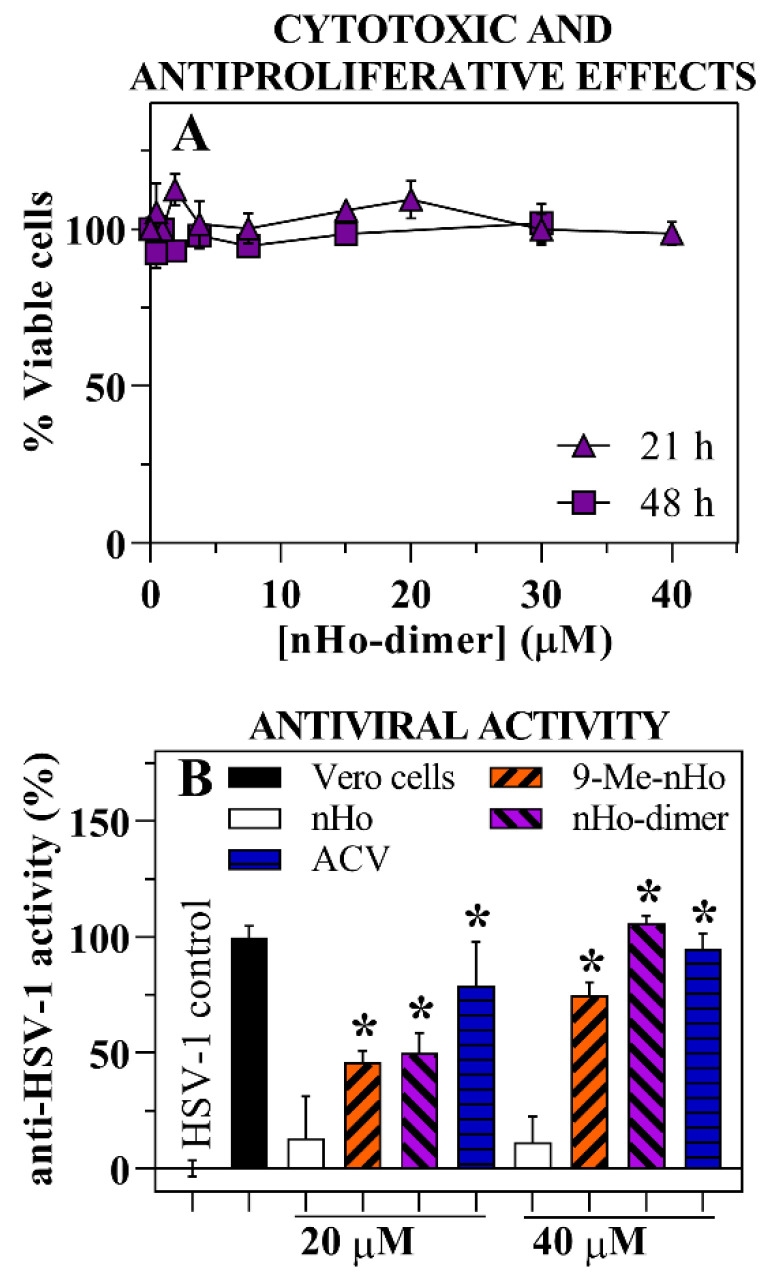

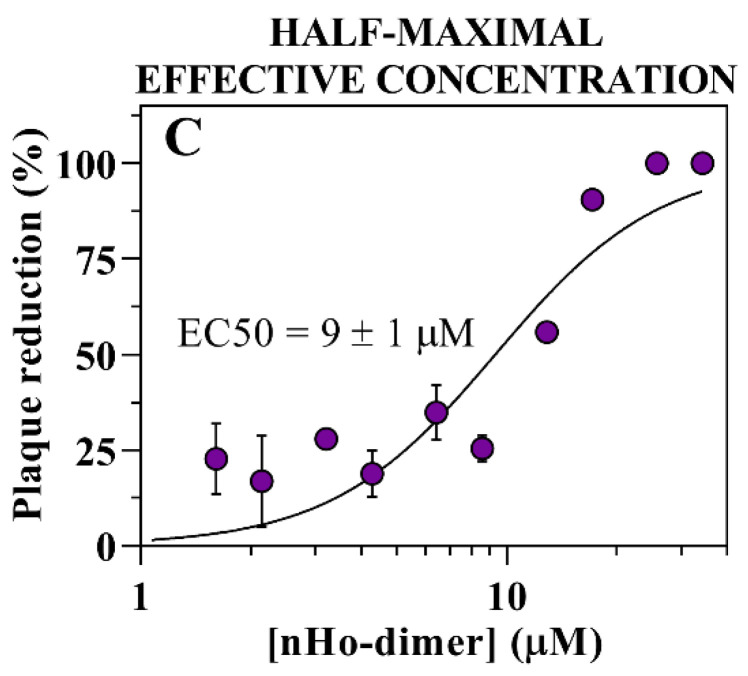
(**A**) Cytotoxic and antiproliferative effects of nHo-dimer (up to 40 µM) on Vero cells evaluated by MTS assay. Values are means ± SE of triplicates from three independent experiments. (**B**) Percentage of protection against HSV-1 of nHo-dimer (20 and 40 µM) obtained by the MTS assay. Results are means ± SE of triplicates of two independent experiments. (*) indicates significant statistical differences between the tested samples and the HSV-1 control (*p* < 0.05). ANOVA/Dunnett’s tests were carried out. For a comparative purpose, data corresponding to the treatment with ACV and data reported in the literature [15] for two related compounds (nHo and 9-Me-nHo), obtained under the same experimental conditions, were also included. (**C**) Anti-HSV-1 activity at different concentrations, obtained by plaque reduction assays. Values represent the mean ± SE of two independent experiments in triplicate.

To assess the potential cytotoxicity and antiproliferative effects of nHo-dimer, Vero host cells were exposed to varying concentrations of the compound. In order to further differentiate between these processes, two different incubation times were examined (21 h and 48 h), representing a shorter and longer time compared to the cells’ doubling time of 24 h [25]. Due to the low solubility of the compound in the supplemented culture media [26], the maximum concentration tested was 40 µM, preventing the determination of the corresponding CC50 value. Nevertheless, under these experimental conditions, the presence of nHo-dimer did not affect the viability of the host cells (Figure 2A), suggesting a lack of both cytotoxic and antiproliferative effects on the investigated cell line.

### 2.2. Antiviral Effect

Vero cell monolayers infected with HSV-1 were treated with two different concentrations of nHo-dimer (20 and 40 µM). In both of these conditions, the dimer demonstrated a potent antiviral effect, providing approximately 50% and 100% protection, respectively, which was comparable, albeit slightly lower, than the protection conferred by ACV tested under identical experimental conditions (Figure 2B). Microscopic inspection revealed that nHo-dimer prevented signs of virus-driven cytopathic effects (i.e., the appearance of cytoplasmic vacuoles and cells rounding up). The antiviral activity of nHo-dimer was further evaluated and confirmed by plaque reduction assays. The dose-dependent reduction in viral plaques yielded an EC50 value of 9 (±1) µM (Figure 2C).

### 2.3. Effect on the Virus Life Cycle and Its Potential Mechanisms of Action

**Figure 3:**
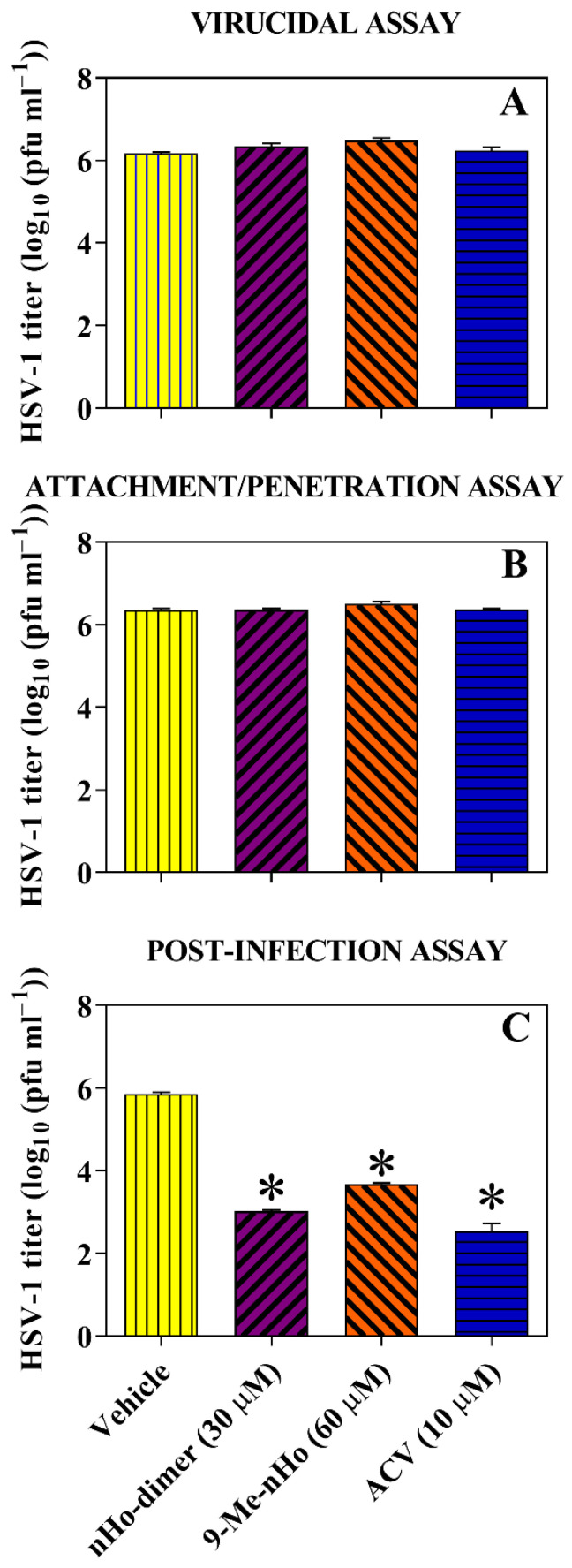
Evaluation of effects of nHo-dimer (30 µM) at three different levels or stages of HSV-1 infection: (**A**) virucidal assay, (**B**) attachment/penetration assay and (**C**) post-infection assay. Bars are means ± SE of viral titers, obtained by titration of cell lysates (duplicates), and they are representative of two independent experiments with similar results. Statistical differences (*p* < 0.05) are indicated as (*). ANOVA/Dunnett’s tests were performed for each compound and compared to the respective controls. For comparative reasons, results for 9-Me-nHo (60 µM) and ACV (10 µM), obtained under identical experimental conditions reported in the literature [15], are included.

The effect of nHo-dimer on both HSV-1 and HSV-2 was evaluated at three different stages: ***(i)*** its potential *virucidal effect* was evaluated by pre-incubating extracellular infectious viral particles of HSV-1 or HSV-2 with nHo-dimer (30 µM) during 1 h. After this incubation time, the host cells were infected with treated viruses. No virucidal effect was observed, as the treated viral particles infected the cells in the same manner as the untreated ones (Figure 3A and Supplementary Figure S1A). ***(ii)*** To evaluate a possible *prophylactic activity*, the cell monolayers were pre-treated with nHo-dimer during 1 h before virus infection. The results indicate that this pre-treatment did not activate anti-HSV mechanisms in the host cells, nor did it interfere with viral adsorption/penetration steps (Figure 3B and Supplementary Figure S1B). ***(iii)*** Additionally, the *post-infection* treatment with nHo-dimer was assessed. Our data demonstrate that the dimer caused a significant reduction in virus titers upon addition to infected cells (Figure 3C and Supplementary Figure S1C). This suggests that nHo-dimer remains active during viral replication within the host cell.

**Figure 4:**
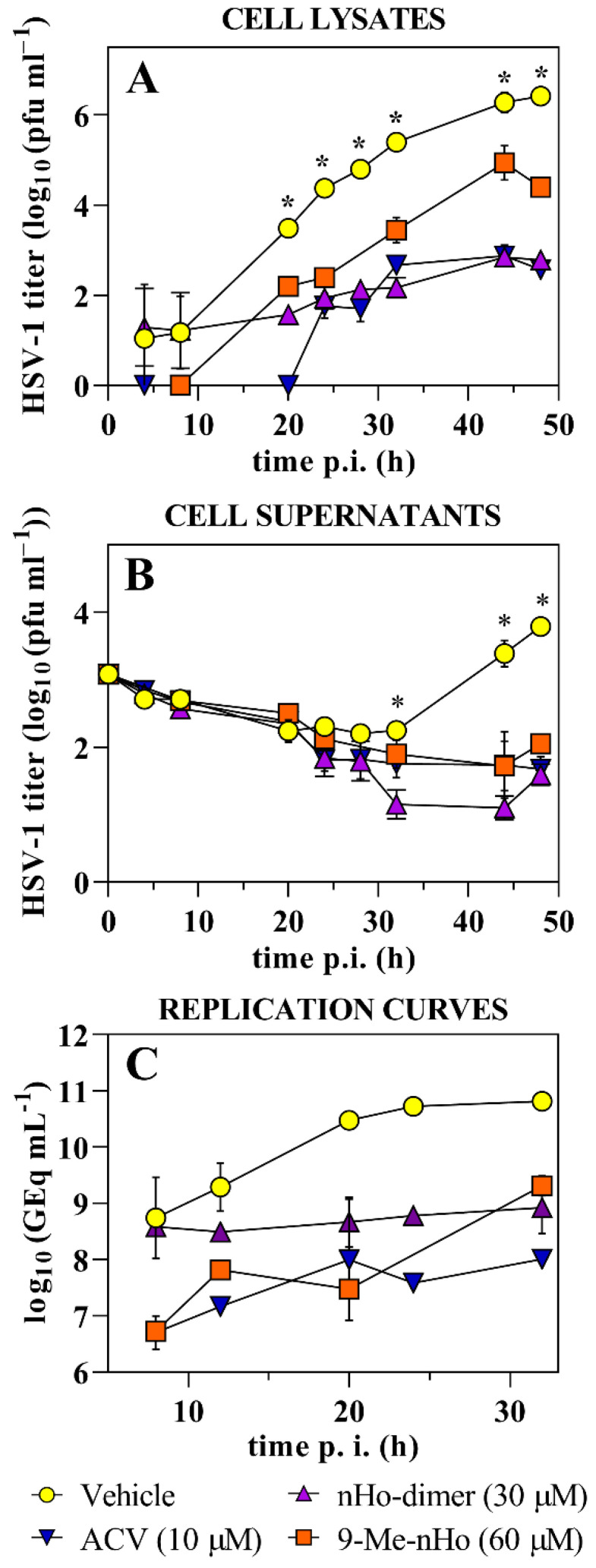
HSV-1 yields in the presence of nHo-dimer (30 µM) or the vehicle. The values were obtained by means of virus titration of cell lysates (**A**) or cell supernatants (**B**) and represent the mean of duplicates (±SD). * indicates significative differences between samples treated with nHo-dimer or the vehicle (*p* < 0.05, multiple *t*-test/Holm–Sidak method). (**C**) Viral replication curves where data represent the mean of the number of HSV-1 genome copies (±SD) obtained by quantitative PCR after treatment with nHo-dimer (30 µM) at different times. For comparative reasons, data reported for 9-Me-nHo (60 µM) and ACV (10 µM) are included [15].

The HSV-1 and HSV-2 yield reduction was evaluated at different infection times (Figure 4 and Supplementary Figure S2, respectively). Briefly, in the case of HSV-1 at early stages of infection (8 h.p.i), the dimer did not inhibit viral replication. However, from 20 h.p.i., the virus yield showed a significant reduction when compared with untreated viral particles. This is even more evident at longer infection times (Figure 4A). Furthermore, the release of infective HSV-1 particles to the medium was decreased in the presence of nHo-dimer (Figure 4B). For HSV-2, although it was with a less pronounced effect in all treatments, the virus yield reduction showed a similar trend to HSV-1 (Supplementary Figure S2A,B). In this case, the massive lysis of the control monolayers differed in 14 h with the monolayers treated with nHo-dimer (~30 h and 44 h after incubation, respectively).

The replication curves (Figure 4C) in the presence of nHo-dimer (30 µM) followed a similar trend to that observed for the experiments of virus yield reductions (Figure 4A). HSV-1 genome equivalents/mL remained practically at the same level in contrast to the genome copies of the control samples, which increased by 2 log units at time 32 h.p.i. Although other mechanisms of action could explain the latter results, one putative explanation could be that the nHo-dimer may also affect viral DNA replication. This should be further explored.

**Figure 5:**
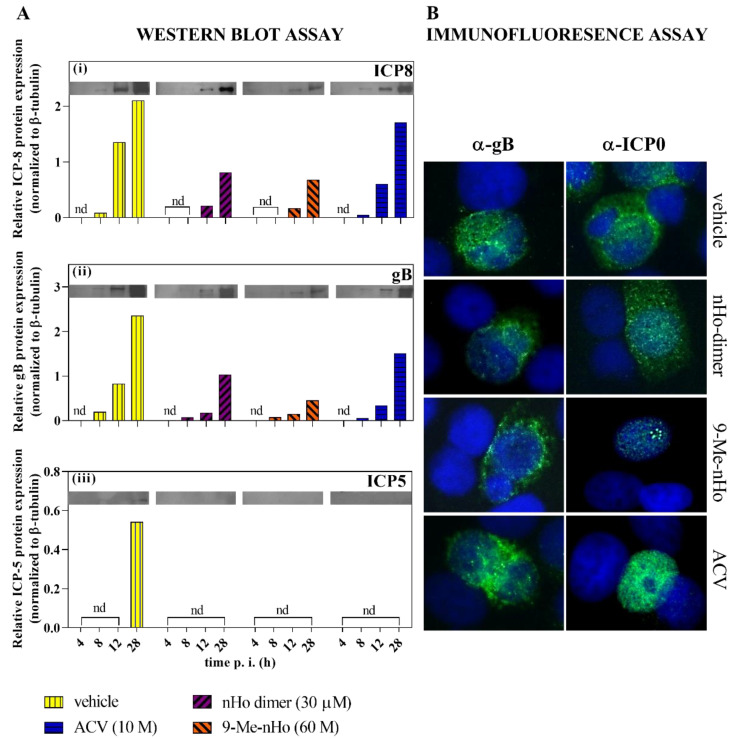
(**A**) Western blots and the semiquantitative analysis (relative to β-tubulin) of HSV-1 early (ICP8) and late (gB, ICP5) proteins, respectively, in the presence of nHo-dimer (30 µM) evaluated at 4, 8, 12 and 28 h.p.i. For comparative reasons, data reported for 9-Me-nHo (60 µM) and ACV (10 µM) are also included. (**B**) Immunofluorescence microscopy examining ICP0 and gB subcellular localization (in green) after 20 h of treatment with nHo-dimer (30 µM). DAPI (in blue) was used for nuclear staining. For comparative reasons, results for 9-Me-nHo (60 µM) and ACV (10 µM), obtained under identical experimental conditions and previously reported in the literature, are included. Erratum: note that, by mistake, the labels of images depicted in the original publication [15] are inverted.

The replication cycle of herpes viruses is conventionally separated into three temporal phases, named immediate–early (IE), early (E) and late (L), in which the early-expressed proteins include transcription factors and/or modulators of host immune responses and cell environment, and the late-expressed ones form structural components of the virion [27]. To delve into the stage of the viral cycle altered by nHo-dimer, the expression levels of HSV-1 early (ICP8) and late (gB, ICP5) proteins were analyzed by Western blot (Figure 5A). When comparing these results, the expression of ICP8 was noticeable at 8 hpi in cells treated with ACV and in the untreated control group. However, this expression was negligible in cells treated with the nHo-dimer and 9-Me-nHo. By 12 hpi and 28 hpi, ICP8 expression was higher in the control group compared to cells treated with either compound. At 28 hpi, there was a substantial reduction in ICP8 expression levels in infected cells treated with both βCs. A similar pattern was observed for gB, whereas the major capsid protein ICP5, which was expressed with late kinetics, was only noticeable in the untreated control at 28 hpi. On the contrary, infected cells treated with nHo-dimer showed a clear inhibition of ICP5 expression. In addition, the effect of nHo-dimer on the intracellular localization of the immediate–early (IE) HSV E3 ubiquitin ligase ICP0 (or infected cell polypeptide 0) in infected cells was evaluated by fluorescence microscopy (Figure 5B). Immunofluorescence images showed that the presence of nHo-dimer on infected cells had no effect on the cytoplasmic localization of ICP0 in contrast to the monomer 9-Me-nHo, described elsewhere [15], that somehow retained the protein ICP0 in the nucleus.

### 2.4. Cellular Uptake

**Figure 6:**
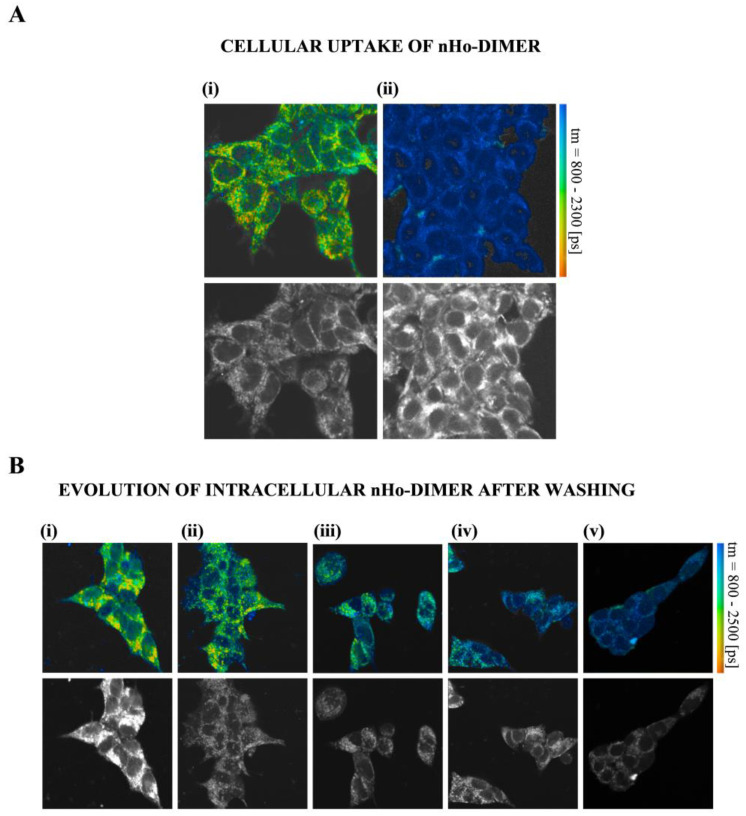
(**A**) (**i**) Representative fluorescence lifetime (colored) and intensity (black and white) images of HEK293 cells incubated for 30 min with nHo-dimer (30 μM), recorded under two-photon excitation at a wavelength of 850 nm using bp allvis emission filter. The mean value of average fluorescence lifetime (μ) was 1.5 ns. Column (**ii**) depicts a representative fluorescence image of wt HEK293 cells in the absence of nHo-dimer. The mean value of average fluorescence lifetime (μ) was 0.7 ns. (**B**) Columns (**i**–**iv**) depict fluorescence images of HEK293 cells incubated for 30 min with nHo-dimer (30 μM) and then washed with fresh media, recorded every 5 min. The mean value of average fluorescence lifetime (μ/ns) changed from (**i**) 1.45, (**ii**) 1.28, (**iii**) 1.14 and (**iv**) 0.98. Column (**v**) depicts a representative fluorescence image of wt HEK293 cells in the absence of nHo-dimer (autofluorescence, μ = 0.89).

The cellular internalization of nHo-dimer on HEK293 cells was characterized by two-photon excitation laser-scanning fluorescence microscopy coupled to fluorescence lifetime imaging (FLIM) (Figure 6). The two related monomers, 9-Me-nHo and 9-Me-Ho, were also investigated (Supplementary Figures S3–S5). In all cases, a clear internalization of the three βCs was observed in FLIM images with two-photon excitation at 840 or 850 nm, for the 9-Me-βCs and the dimer, respectively. Untreated HEK293 cells showed a multiexponential (three-exponential or more) fluorescence decay with short average fluorescence lifetimes (mean value well below 1 ns (Figure 6(Aii,Bv), and Supplementary Figures S3(Aii,Bii), S4v and S5v)). On the contrary, cells that had been incubated with nHo-dimer for 30 min showed significantly prolonged fluorescence decays in all parts of the cell, most prominently in the perinuclear region (Figure 6A). The mean of the average fluorescence lifetime was increased to more than 1.5 ns. Similar behavior was observed for 9-Me-nHo and 9-Me-Ho (Supplementary Figures S3, S4i and S5ii).

The presence of the βCs in the external medium during the microscopic measurements led to strong cellular uptake. As a typical example, we showed HEK293 cells loaded with 9-Me-Ho (Figure S5i), while the βC is present in the extracellular medium (50 µM). The average fluorescence lifetime was greatly enhanced (almost 3 ns) [28,29] and the fluorescence intensity was three to four times larger compared to that of 9-Me-Ho in the extracellular solution, proving an enrichment of the compound in the cells.

Interestingly, after washing the cells with fresh external media free of βCs, the respective signals corresponding to the three βC alkaloids were partially lost on a minute time scale (Figure 6B and Supplementary Figures S4 and S5). This phenomenon was previously described for other related βCs, and it is compatible with a passive (diffusional) uptake mechanism where enhanced permeability is expected for the neutral βC derivatives [28]. The images indicate that the three compounds investigated localized to the same intracellular structures, including perinuclear organelles (predominantly mitochondria), as well as intranuclear structures. This observation aligns with previous findings reported for 9-Me-nHo [28].

## 3. Discussion

The current pharmacological treatments against HSV infection have clinical limitations, mainly because of the resistant variants arising from long-term therapies needed in immunocompromised patients [1,30]. Hence, finding novel and safe anti-HSV drugs is needed. Monomeric βC alkaloids have proven to exert in vitro and in vivo actions against these viruses [15,16,17]. Thus, βCs could serve as potential candidates as non-nucleotide antiherpetic agents, exerting a different or complementary mechanisms of action than acyclovir (ACV), the gold standard in the treatment of herpes virus infections.

Among others, βC’ dimers have not yet been deeply investigated for their antiviral capability. In this context, an in vitro study of the effect of the 9,9′-norharmane dimer against HSV is reported herein. Anti-HSV-1 screening showed different behavior when comparing the dimer with the respective N(9)-unsubstituted monomer (nHo). Briefly, under the same conditions, the monomer was inactive, whereas nHo-dimer exerted a significant protection (Figure 2B). This is consistent with previous reports that the substitution at position N(9) enhances anti-HSV-1 activity [15]. On the other hand, dimers have been shown to exert higher antitumoral activities than monomers on tumoral cell lines [7] and better antiviral properties than monomers against pseudorabies virus (PRV) [31]. Plaque reduction assays confirmed the anti-HSV-1 activity of the dimer, with an EC50 of 9 ± 1 µM (Figure 2C), which is slightly higher than the EC50 previously reported for 9-Me-nHo (4.9 ± 0.4 µM). These results, together with the similar behavior that both compounds showed in the MTS screening, strongly suggest that the enhancement of the antiviral activity is related more to N(9)-substitution than to the bivalent action of the dimer. Furthermore, several in vitro studies about the potency of different related βC dimers as anticancer agents, showed that a spacer of 4-6 methylene units is needed to achieve a bivalent effect [26]. Also, the anti-PRV activity of several bivalent 9-methyl-harmine compounds, with linkers containing four to eight methylene units, decreases with the prolongation of the linker [31]. Nevertheless, according to our previous studies reporting the antiviral activity of several βCs (particularly 9-Me-nHo) against HSV-1 [15] and the results obtained herein for the dimer, a multifactorial mechanism of action can be suggested.

The cytotoxicity of nHo-dimer was tested on the host cells to rule out the concept that the observed antiviral effect could have been due to a loss of cell viability. In the concentration range (up to 40 µM), the dimer did not show any cytotoxic or antiproliferative effect (Figure 2A). This is in agreement with the fact that N(9)-methyl substitutions of the βC monomers might have a protective effect, reducing, in some cases, the overall cytotoxicity [15,21]. Even though the corresponding CC50 value for the dimer was not determined due to its low solubility in culture media, based on the reported data for the norharmane monomer (nHo) and the two related N(9)-methyl-derivatives, 9-Me-Ho and 9-Me-nHo, we can infer that the dimer would exhibit low cytotoxicity (CC50 >> 200 μM) [15]. Despite this, when considering 30 μM as a lower limit for CC50, together with the EC50 value reported in the previous paragraph, an underestimated selective index or SI (i.e., the ratio that measures the window between cytotoxicity and antiviral activity) larger than 3 can be calculated in the case of the dimer.

Time of addition experiments performed with HSV-1 (Figure 3) and HSV-2 (Supplementary Figure S1) showed that nHo-dimer is not virucidal. Several reports also describe the lack of virucidal activity of different compounds in this family against HSV-1 [15,16], HSV-2 [15,17] and other viruses, such as PV [19] or DENV-2 [21]. The attachment and penetration steps are not affected by nHo-dimer either. Instead, the dimer is active after the viral infection of the host cell. It is noteworthy that, as demonstrated in these results and those from virus yield assays (Figure 4 and Supplementary Figure S2), this compound shows a trend similar to that observed for other related monomeric βCs previously investigated, including 9-Me-nHo [15]. Additionally, fluorescence lifetime imaging progressing with time indicates the partial loss of βCs in intracellular compartments after washing (Figure 6B and Supplementary Figures S4 and S5), which could explain the absence of a prophylactic effect exhibited by these compounds. Therefore, the antiviral action of these alkaloids appears to be dependent on their continuous presence in the incubation media.

Some βC-monomers have been shown to exert part of their antiviral activity through decreasing immediate–early (IE), early (E) and late (L) protein expression. For example, harmine suppresses the HSV-1 mRNA expression of the IE genes codifying for ICP0, ICP4 and ICP27 and also reduces the expression of ICP5 in HSV-1 and HSV-2 in a dose-dependent manner [17]. Harmaline, the harmine 3,4-dihydro analog, also decreases HSV-1 ICP4 and ICP27 levels and the expression of both transcripts in a time-dependent manner [16]. In this work, we have investigated the effect of nHo-dimer on ICP8, ICP5 and gB expression (Figure 5A). The first one is a multifunctional single-stranded DNA-binding protein, which is essential for viral DNA replication mediated by its annealing activity [32]. ICP5 is a major structural protein which acts as a scaffold during the formation of HSV’s capsid and plays an important role in virus packaging and maturity. This protein is necessary for HSV-1 proliferation [33]. Finally, gB is an envelope glycoprotein required for HSV fusion [34]. Our previous results showed that 9-Me-nHo, 9-Me-Ho and 6-methoxyharmane strongly reduce the level of expression of ICP8, ICP5 and gB [15]. As the expression of HSV proteins follow a cascade of temporal events, this fact could be caused by the inhibition of IE events, as other authors have previously shown [16,17]. A similar pattern in the ICP8, gB and ICP5 protein expression was found for nHo-dimer.

Host cells control viral infections through antiviral cellular responses triggered by pathogen-associated molecular patterns [35,36]. This process, mediated in part by Toll-like receptors, leads to the activation of intracellular signaling pathways that induce the expression and secretion of molecules that limit viral replication, alert neighboring cells, and activate the immune system [37,38,39]. In return, the viral genome encodes for effectors that act by either altering repressive complexes or degrading key restrictive factors. For example, ICP0, a multifunctional IE protein that works as a transactivator for IE, E and L viral genes, determines the balance between lytic replication and reactivation from latency but also targets host defensive molecules for ubiquitin-mediated proteasomal degradation [40,41,42]. These characteristics make ICP0 an attractive target for HSV-1 antiviral therapy. During infection, ICP0 changes its location. Initially, its nuclear localization signal (NLS) guides it to the nucleus, where it localizes to nuclear domain 10 (ND10), a dynamic structure involved in antiviral defense. One of the ICP0 nuclear functions involves dispersing ND10 bodies. Subsequently, ICP0 diffuses throughout the nucleus [43], and when its nuclear functions are completed [44], it relocates to the cytoplasm, where it plays roles in suppressing type I IFN-mediated antiviral effects and activating the NF-κB pathway [41]. Although some aspects of its movement from the nucleus to the cytoplasm are not fully understood, the C-terminal 35 amino acids motif in ICP0 is essential for this process [45]. It has also been reported that the cytoplasmic translocation of ICP0, required for its incorporation into virions, depends on ICP27 [46]. Additionally, late viral proteins might assist in this translocation, as hindering viral DNA synthesis delays ICP0’s translocation [47,48]. Furthermore, a defective ubiquitination and the inhibition of cyclin-dependent kinase 4 (cdk4) [49] or the inhibition of histone deacetylases (HDACs) [43] sequester ICP0 in the nucleus. Interestingly, at very early stages of the infection, harmaline interferes with the binding of the IE complex to the ICP0 promoter [16], and several βCs can inhibit cdk4 [50] and HDACs [51]. These facts led us to also investigate the localization of ICP0 in the presence of nHo-dimer. Our previous results demonstrated that some monomeric βCs, such as 9-Me-nHo, change the localization pattern of ICP0 at late stages of the infection (Figure 5B) [15]. Specifically, ICP0 remained in the nucleus, forming small foci, consistent with co-localization with ND10, similar to early HSV-1 infection [45]. This aligns with the strong inhibition of E and L protein expression caused by 9-Me-nHo, as late viral proteins typically facilitate ICP0’s cytoplasmic translocation. However, the dimer did not affect the normal cytoplasmic translocation of ICP0. As the dimer does not inhibit the expression of L proteins (gB) as effectively as 9-Me-nHo, this may not be sufficient to alter the usual localization pattern of ICP0. If monomeric βCs are also implicated in the additional mechanisms that sequester ICP0 in the nucleus, should be the object of further studies.

## 4. Materials and Methods

### 4.1. Chemical and Reactants

The non-commercial β-carbolines (βCs, purity > 98%) used in this study, namely, nHo-dimer, 9-Me-nHo and 9-Me-Ho, were synthesized and fully characterized by ^1^H- and ^13^C-NMR, FTIR, microanalysis, EI-MS, UV-vis spectroscopy as well as DSC, TGA, HRESI-MS, and fluorescence spectroscopies, among others, following the procedures described elsewhere [29,52].

#### Preparation of Stock Solutions

βCs’ stock solutions (with concentrations ranging from 1.5 to 2 mM, depending on the solubility of the compounds) were prepared by dissolving the neutral alkaloid in pH 3 sterile ultrapure water [15,53]. Subsequently, these neutral stock solutions were further diluted to working concentrations (2% *v*/*v*) in either Dulbecco’s Modified Eagle Medium (DMEM) supplemented as described below or PBS for antiviral or microscopy experiments, respectively (final pH = 7.2–7.4).

### 4.2. Virus and Cell Cultures

The methods for virus and cell culture largely follow the procedure outlined by [15] with slight modifications required for this study.

#### 4.2.1. Cell Culture

Vero cells were used in this study and were cultured in Dulbecco’s Modified Eagle’s Medium (DMEM) supplemented with 5% fetal bovine serum, 1% L-glutamine, 1% non-essential amino acids, 1% penicillin/streptomycin, 0.1% gentamycin and 0.2% fungizone (Life Technologies, Munich, Germany) [15].

#### 4.2.2. Virus Propagation

Two clinical isolates of HSV-1 and HSV-2 (identity confirmed by sequencing at the Max-von-Pettenkofer Institute, LMU, Munich, Germany) were employed. The viruses were propagated in Vero cells. To determine the amount of viral particles in the stocks, plaque assays were conducted following the protocol described elsewhere [54]. Briefly, Vero cell monolayers grown on 24-well plates were infected with serial dilutions of viral stocks, centrifuged at RT (500 rpm, 5 min) and incubated at 37 °C in a 5% CO_2_ humidified atmosphere for 1 h. Subsequently, supernatants were removed, and infected cells were incubated in DMEM with 0.75% carboxymethylcellulose added (Sigma, Munich, Germany) during 72 h. Then, the cell monolayers were fixed and stained with 0.1% *p*/*v* of crystal violet in 20% *v*/*v* ethanol in sterile ultrapure water. Viral stocks were stored at −80 °C.

### 4.3. Cytotoxicity Assays

#### 4.3.1. MTS Assay

Vero cell monolayers, cultured in 96-well microplates until they reached 90% confluence, were exposed to different concentrations of nHo-dimer diluted in DMEM, as described in Section 4.1. The cytotoxicity of nHo-dimer was evaluated using the MTS colorimetric assay [55] at two time points (21 and 48 h) using the CellTiter 96^®^ AQueous One Solution Reagent (Promega, Madison, WI, USA). The negative control group was treated with the vehicle only.

#### 4.3.2. Calculation of Cell Viability

The absorbance (*A*^490nm^) of each well was measured using a microplate reader (Tecan, Sunrise^TM^, Grödig, Austria). The percentage of cell viability was calculated using the following equation:

(1) $$\%\;cell\;viability=\;\frac{\left(A490\;sample-A490\;background\right)}{(A490\;cell\;control-A490\;background}\times 100$$

where *A*^490^_*sample*_ is the absorbance of each sample well containing cells treated with nHo-dimer, *A*^490^_*cell control*_ is the average absorbance of wells containing cells treated only with the vehicle (negative control) and *A*^490^ *_background_* is the average absorbance of wells containing the reagent without cells. Results were obtained in triplicate in two independent experiments.

### 4.4. Evaluation of Antiviral Activity

The antiviral activity of nHo-dimer was carried out following a modified cytotoxicity protocol outlined in Section 4.3, as it was described elsewhere [15]. Briefly, Vero cell monolayers grown in 96-well microplates were treated with 20 or 40 µM concentrations of nHo-dimer or the vehicle (control group), and were infected with HSV-1 at a viral load of 100 PFU/well. Following infection, the microplates were incubated until cytopathic effects were evident in the control cultures (96 h). Then, the culture medium was replaced, and the MTS assay was performed in triplicate. To quantify the antiviral activity, the percentage of inhibition was calculated using the following equation:

(2) $$\%\;antiviral\;activity=\frac{\left(A490\;sample-A490\;virus\;control\right)}{\left(A490\;cell\;control-A490\;virus\;control\right)}\times 100$$

where *A*^490^_*virus control*_ represents the average absorbance measured from three wells containing virus controls (cells infected with viruses but not treated with drugs). *A*^490^ *_sample_* is the absorbance of each sample well, and *A*^490^ *_cell control_* corresponds to the average absorbance from wells containing cell controls (Section 4.3). The antiviral activity of nHo-dimer was assessed in two independent experiments to ensure the reliability and reproducibility of the results.

### 4.5. Plaque Reduction Assays and EC50 Determination

To this aim, the same methodology previously described in the literature [15] was used. Briefly, Vero cells cultured in standard medium and conditions (Section 4.2.1) were treated with nHo-dimer at different concentrations prior to and during infection with HSV-1. The negative control group was treated with the vehicle only.

#### 4.5.1. Plaque Counting and Plaque Reduction Analysis

After the 48 h incubation period at 37 °C, the cells were fixed and stained with crystal violet. Plaques were manually counted, and the percentage of plaque reduction was determined using the following equation:

(3) $$Plaque\;reduction\;\left(\%\right)=\left(1-\frac{Vd}{Vc}\right)\times 100$$

where *Vd* and *Vc* represent the number of plaques in the presence and in the absence of nHo-dimer, respectively.

#### 4.5.2. EC50 Determination

To determine the effective concentration that results in 50% plaque reduction (EC50), the percentage of plaque reduction was plotted against nHo-dimer concentrations (plaque reduction (%) vs. Log [nHo-dimer]). The data were subject to non-linear regression analysis using the variable Hill’s slope model with GraphPad Prism version 10.2.2 for Windows, GraphPad Software, San Diego, CA, USA, (www.graphpad.com (accessed on 21 February 2024)). Results were obtained in triplicate in two independent experiments. For the following assays, a concentration of nHo-dimer (30 µM) that resulted in 100% plaque reduction was used.

### 4.6. Experiments to Assess the Effect of nHo-Dimer during Different Stages of Infection. Time-of-Addition Experiments

The experimental design for assessing the effects of nHo-dimer during various stages of infection followed the methodology reported elsewhere [15], which is briefly outlined below.

#### 4.6.1. Virucidal Activity Assay

Suspensions of HSV-1 and HSV-2 (2000 PFU/mL) were separately treated with nHo-dimer (30 µM) or the vehicle at room temperature (RT) for 1 h. Subsequently, treated viruses were added to Vero cell monolayers cultivated in 12-well plates. The cell–virus mixtures were then centrifuged at 500 rpm during 5 min and incubated during at 37 °C for 1 h to facilitate virus uptake. After incubation, the medium was aspirated, and the cells were washed once with PBS. The plates were further incubated in fresh DMEM prior to the extensive cytopathic effect being observed in the control cultures (48 h and 28 h for HSV-1 and HSV-2, respectively). Harvested cells were subjected to freeze–thaw cycling to lyse the cells, and the resulting infectious viral particles were titrated on Vero cells.

#### 4.6.2. Assessment of Viral Attachment

To determine whether the βC interfered with the attachment or penetration stages of viral infection, Vero cell monolayers grown in 12-well plates were incubated at 37 °C for 1 h, with DMEM supplemented with nHo-dimer (30 µM) or the vehicle. Cells were washed with PBS, and HSV-1 or HSV-2 (2000 PFU/well) was added to the cells. As described in 4.6.1, the plates were incubated for 48 and 28 h for HSV-1 and HSV-2, respectively. Cells were then harvested and lysed by cycles of freezing and thawing, and the infectious viral particles were titrated on Vero cells.

#### 4.6.3. Post-Infection Treatment

To explore whether nHo-dimer influences viral replication, Vero cell monolayers were infected with HSV-1 or HSV-2 (2000 PFU/well). The plates were then centrifuged at 500 rpm for 5 min and incubated at 37 °C for 1 h to allow virus uptake. After the incubation period (48 h and 28 h for HSV-1 and HSV-2, respectively), the cells were washed once with PBS, and fresh DMEM supplemented with nHo-dimer (30 µM) or the vehicle was added. The subsequent procedures were performed following the protocol described in Section 4.6.1. This set of assays was conducted in duplicate and repeated, at least, in two independent experiments.

### 4.7. Effect of nHo-Dimer on Viral Replication

Vero cells cultured to confluency in 12-well plates were treated with a concentration of 30 µM nHo-dimer or the corresponding vehicle (control) before and during the infection with HSV-1 or HSV-2, at a viral titer of 2000 PFU/well. At different time points post-infection, the culture supernatants and cells were collected separately. The viruses were quantified using plaque assays performed in duplicate, as reported elsewhere [15].

### 4.8. Quantitative PCR (qPCR)

Cell monolayers infected with HSV-1 were incubated with nHo-dimer (30 µM) or the vehicle during different times. Subsequently, the cells were harvested for further qPCR analysis. Nucleic acids were extracted from the harvested cells using the MagNA Pure LC 2.0 System (Roche Diagnostics GmbH, Penzberg, Germany), and qPCR was performed on the extracted DNA, following established protocols [15]. Briefly, specific HSV-1 sequences were amplified from 5 µL of extracted DNA using a 7500 Fast Real-Time PCR System and TaqMan^®^ Fast Universal PCR Master Mix (Thermo Fisher Scientific Inc., Waltham, MA, USA). To amplify a specific 81-nt sequence of the gene encoding the HSV-1 polymerase, we employed optimized primers and a FAM-labeled probe. The thermocycler settings for PCR amplification were as follows: initial denaturation at 95 °C for 20 s, followed by 45 cycles of denaturation at 95 °C for 3 s, and annealing/extension at 60 °C for 30 s. Quantification was accomplished by the simultaneous amplification of four HSV-plasmid-DNA standards of 5 × 10^2^, 5 × 10^3^, 5 × 10^4^ and 5 × 10^5^ copies/mL.

### 4.9. Immunoblotting

Immunoblotting was employed to investigate the protein expression levels in HSV-1-infected cells treated with nHo-dimer (30 µM) or the vehicle, following a previously described protocol [15]. Briefly, the whole-cell protein extracts were prepared and separated using SDS-PAGE. The proteins were transferred onto nitrocellulose membranes. The membranes were blocked with 5% skim milk in a buffer containing 20 mM Tris (pH 7.5), 150 mM NaCl and 0.5% Tween 20, followed by rinsing and incubation with specific monoclonal antibodies targeting ICP8 (Sta. Cruz, CA, USA), β-tubulin, ICP5 and gB (all provided by Abcam, Boston, MA, USA), for 3 h at room temperature. After rinsing, the membranes were exposed to HRP-labeled anti-mouse antibodies in 1X TBST for 1 h at room temperature, and protein bands were visualized using an ECL Western blot detection kit (G&E, Buckinghamshire, UK).

### 4.10. Immunofluorescence Staining and Microscopy

Herein, we used the same protocol described elsewhere [15]. Vero cell monolayers were cultured in 24-well plates and infected with HSV-1 (1000 PFU/well). To assess the impact of nHo-dimer, cells were treated with 30 µM of nHo-dimer or the vehicle control. After 20 h of incubation, the cell culture medium was removed, and the cells were washed once with PBS before fixation with 4% paraformaldehyde for 30 min. Following fixation, cells permeabilization was achieved using 0.5% Triton X-100 (Sigma-Aldrich, Munich, Germany), and non-specific binding was blocked using 10% *v*/*v* goat serum and 0.3% Triton X-100 in PBS. Then, cells were incubated for 3 h at room temperature with monoclonal anti-ICP0 (Sta. Cruz, CA, USA) and anti-gB (Abcam, Boston, MA, USA) antibodies. Nuclei were stained with DAPI (Sigma-Aldrich, Munich, Germany).

### 4.11. Other Relevant Information

The experiments outlined in Section 4.3, Section 4.4, Section 4.5, Section 4.6, Section 4.7, Section 4.8, Section 4.9 and Section 4.10 were conducted at a Multiplicity of Infection (MOI) of 0.006. In the statistical analysis, data were analyzed by one-way ANOVA, followed by Dunnett’s post-test (after checking for normality and homoscedasticity) or by multiple *t*-test without assuming consistent SD and corrected for multiple comparisons using the Holm–Sidak method. Results were considered significant at *p* < 0.05. GraphPad Prism 8.0.2 was used to this end.

### 4.12. Fluorescence Lifetime Imaging Microscopy (FLIM) in Living Cells

#### 4.12.1. Fluorescence Lifetime Images

Fluorescence lifetime images were acquired using an upright laser-scanning fluorescence microscope (LSM880 microscope, Zeiss, Jena, Germany) with a 20× water immersion objective (numerical aperture 1.0, working distance 2.1 mm, XYZ Zeiss). Fluorescence was excited by 2-photon excitation using 120 fs light pulses with either λ_exc_ = 840 nm or 850 nm. Laser pulses were generated using a mode-locked Titan-Sapphire laser (InSight X3, Newport Spectra Physics, Darmstadt, Germany), with output powers ranging from 1.9 to 2.3 W for different excitation wavelengths, at a repetition rate of 80 MHz, while only a small fraction (typical: 10 mW) was applied to the sample.

#### 4.12.2. Fluorescence Lifetime Determination

Mean fluorescence lifetimes were determined in the time domain using time-correlated single-photon counting (TCSPC), with dedicated electronics and acquisition software (SPC-152, Becker & Hickl, Berlin, Germany) as described elsewhere [56,57,58]. To analyze the fluorescence decays, SPCImage 8.4 software (Becker & Hickl) was used to fit bi-exponential functions to individual fluorescence decays, with a bin size of 2 (averaging the decays of 25 pixels—the central pixel and the surrounding two-pixel layers). The average fluorescence lifetime was determined for each pixel from amplitudes and lifetimes of the two-exponential functions as (a_1_ × τ_1_ + a_2_ × τ_2_)/(a_1_ + a_2_) [58,59].

#### 4.12.3. Incubation of HEK293 Cells with βCs

HEK293 cells (American Type Culture Collection, ATCC CCL-2) were cultured in DMEM containing 10% FBS and 100 μg mL^−1^ of penicillin. A total of 1.5 × 10^4^ cells were seeded in culture dishes and maintained in an incubator at 37 °C with 5% CO_2_ and 95% air for 24 h. Prior to imaging experiments, the culture medium was replaced, and the cells were incubated with 30–50 μM of nHo-dimer, 9-Me-nHo or 9-Me-Ho for 30 min. Control cultures were included in each experiment for comparison.

## 5. Conclusions

In summary, nHo-dimer has antiviral activities against both HSV-1 and HSV-2. This compound, localized in the perinuclear organelles, is not virucidal and does not block virus attachment nor penetration but acts after viral infection. Moreover, one of the mechanisms of antiviral action seems to be mediated by arresting DNA replication. Whether the dimer acts directly or indirectly remains to be further studied. This compound does not show a stronger effect than the monomeric βCs studied previously. In the dimer, the substitution at position N(9) by a voluminous group (compared to a methyl group in 9-Me-nHo) is detrimental regarding the sequestering of ICP0 in the nucleus at the late infection, one important antiviral mechanism of related monomeric βCs. Considering the high efficacy of N(9)-methyl-substituted βCs (including the dimer studied herein), in connection to their distinctive intrinsic mechanisms of action, the use of cocktails of βC alkaloids could be further designed and explored for future treatments to prevent HSV replication. Finally, this is the first report of the anti-HSV activity and mechanism of a structurally conventional βC dimer and lays the groundwork for further research with similar compounds with different N(9)-linker lengths.

## Data Availability

All data generated or analyzed during this study are included in this published article and its Supplementary Information File.

## Supplementary Materials

The following supporting information can be downloaded at: https://www.mdpi.com/article/10.3390/ijms25094966/s1.
