# Supplementary material for: In Vitro Effect of 9,9′-Norharmane Dimer against Herpes Simplex Viruses
Source: Int J Mol Sci. 2024 May 2;25(9):4966. doi: 10.3390/ijms25094966 (PMC11084892; doi:10.3390/ijms25094966)
Supplement: Supplementary file 1 [file ijms-25-04966-s001.zip › ijms-2905658-supplementary.pdf]

Supplementary Material

In Vitro Effect of 9,9′-Norharmane Dimer against Herpes Simplex Viruses

María Micaela Gonzalez [1,2,†], Maria Guadalupe Vizoso-Pinto [3,4,5,†], Rosa Erra-Balsells [6,7], Thomas Gensch [8,*] and Franco M. Cabrerizo [1,2,*]

[1] Instituto Tecnológico de Chascomús (CONICET-UNSAM), Av. Intendente Marino Km 8.2, CC 164 (B7130IWA), Chascomús 7130, Argentina; mgonzalez@intech.gov.ar
[2] Escuela de Bio y Nanotecnologías (UNSAM), San Martín 1650, Argentina
[3] Max von Pettenkofer Institute, Virology, National Reference Center for Retroviruses, Faculty of Medicine, LMU, D-80336 Munich, Germany; mgvizoso@fm.unt.edu.ar
[4] Instituto Superior de Investigaciones Biológicas (INSIBIO), CONICET-UNT, San Miguel de Tucumán 4000, Argentina
[5] Laboratorio Central de Cs. Básicas, Facultad de Medicina, Universidad Nacional de Tucumán, Tucumán 4000, Argentina
[6] Departamento de Química Orgánica, Facultad de Ciencias Exactas y Naturales, Universidad de Buenos Aires, Pabellón II, 3er P., Ciudad Universitaria, Buenos Aires 1428, Argentina; erra@qo.fcen.uba.ar.
[7] Centro de Investigación en Hidratos de Carbono (CIHIDECAR), CONICET, Facultad de Ciencias Exactas y Naturales, Universidad de Buenos Aires, Naturales Pabellón II, 3er P. Ciudad Universitaria, Buenos Aires 1428, Argentina
[8] Institute of Biological Information Processing 1 (IBI-1; Molecular and Cellular Physiology), Forschungszentrum Jülich, Wilhelm-Jonen-Straße, 52428 Jülich, Germany
* Correspondence: t.gensch@fz-juelich.de (T.G.); fcabrerizo@intech.gov.ar (F.M.C.)
† These authors contributed equally to this work.

Contents:

1. Effect of nHo-dimer at different stages of HSV-2. (**Page 3)**
2. HSV-2 yields in the presence of nHo-dimer. **(Page 4)**
3. Cellular internalization of 9-Me-nHo and 9-Me-Ho on HEK293 cells characterized by FLIM. **(Page 5)**
4. Evolution of intracellular 9-Me-nHo fluorescence signal after washing. **(Page 6)**
5. Evolution of intracellular 9-Me-Ho fluorescence signal after washing. **(Page 7)**

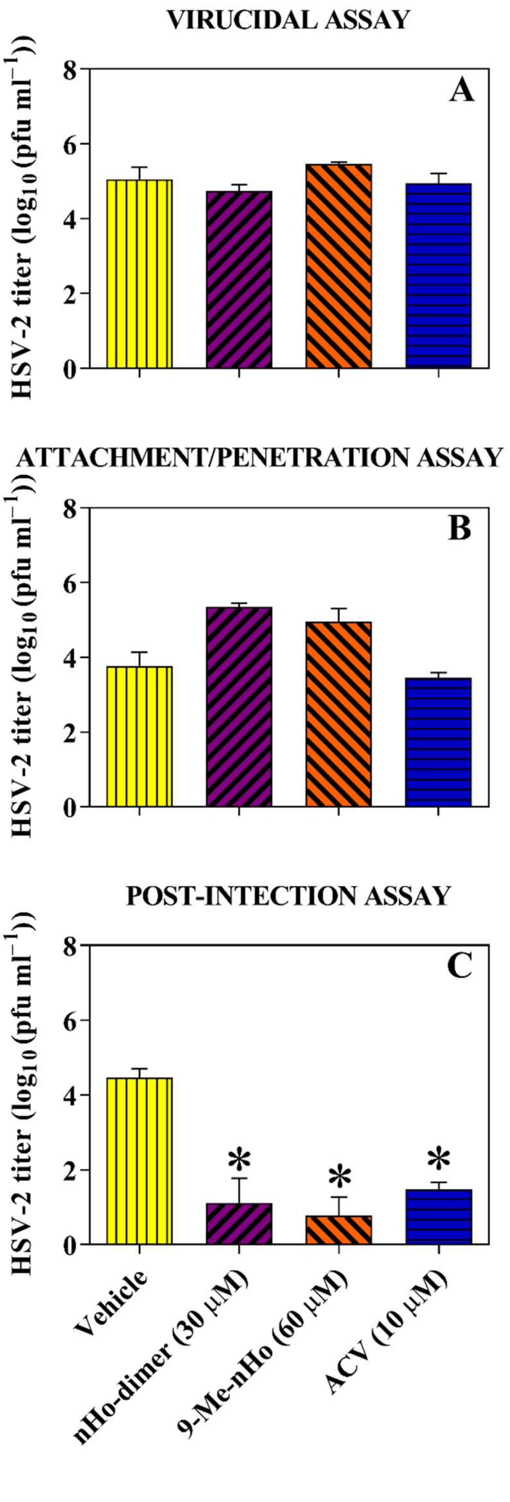

Figure S1. Time of addition experiments to further evaluate the effects of nHo-dimer (30 μM) at three different levels or stages of HSV-2: **A** virucidal assay, **B** attachment/penetration assay, and **C** post-infection assay. Bars are means ± SE of viral titers, obtained by titration of cell lysates (duplicates) and they are representative of two independent experiments with similar results. Statistical differences ($p < 0.05$) are indicated as (*). ANOVA/Dunnett's tests were performed for each compound and compared to the respective controls. For comparative reasons, results for 9-Me-nHo (60 μM) and ACV (10 μM), obtained under identical experimental conditions reported in the literature [15] are included.

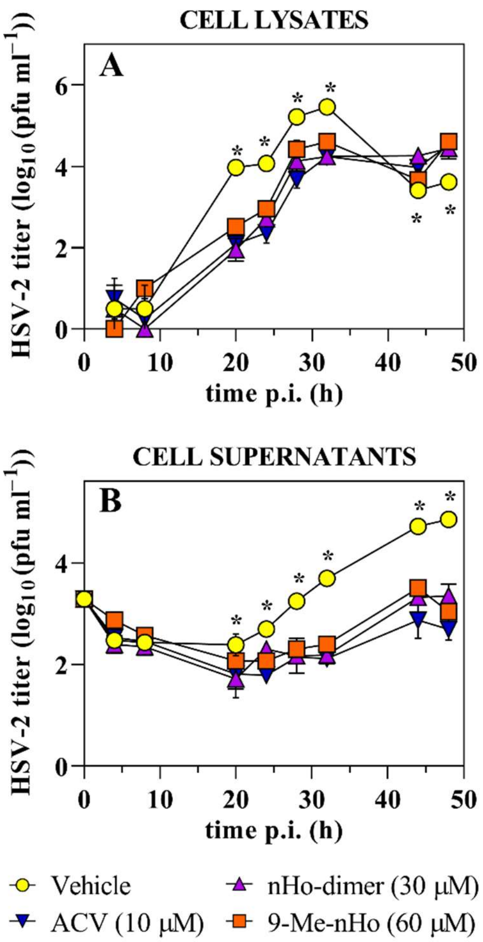

Figure S2. HSV-2 yields in the presence of nHo-dimer (30 μM) or the vehicle. The values were obtained by means of virus titration of cell lysates **(A)** or cell supernatants **(B)** and represent the mean of duplicates (± SD). * indicates significant differences between samples treated with nHo-dimer or the vehicle ($p < 0.05$, multiple t-test / Holm-Sidak method). For comparative reasons data reported for 9-Me-nHo (60 μM) and ACV (10 μM) are included [15].

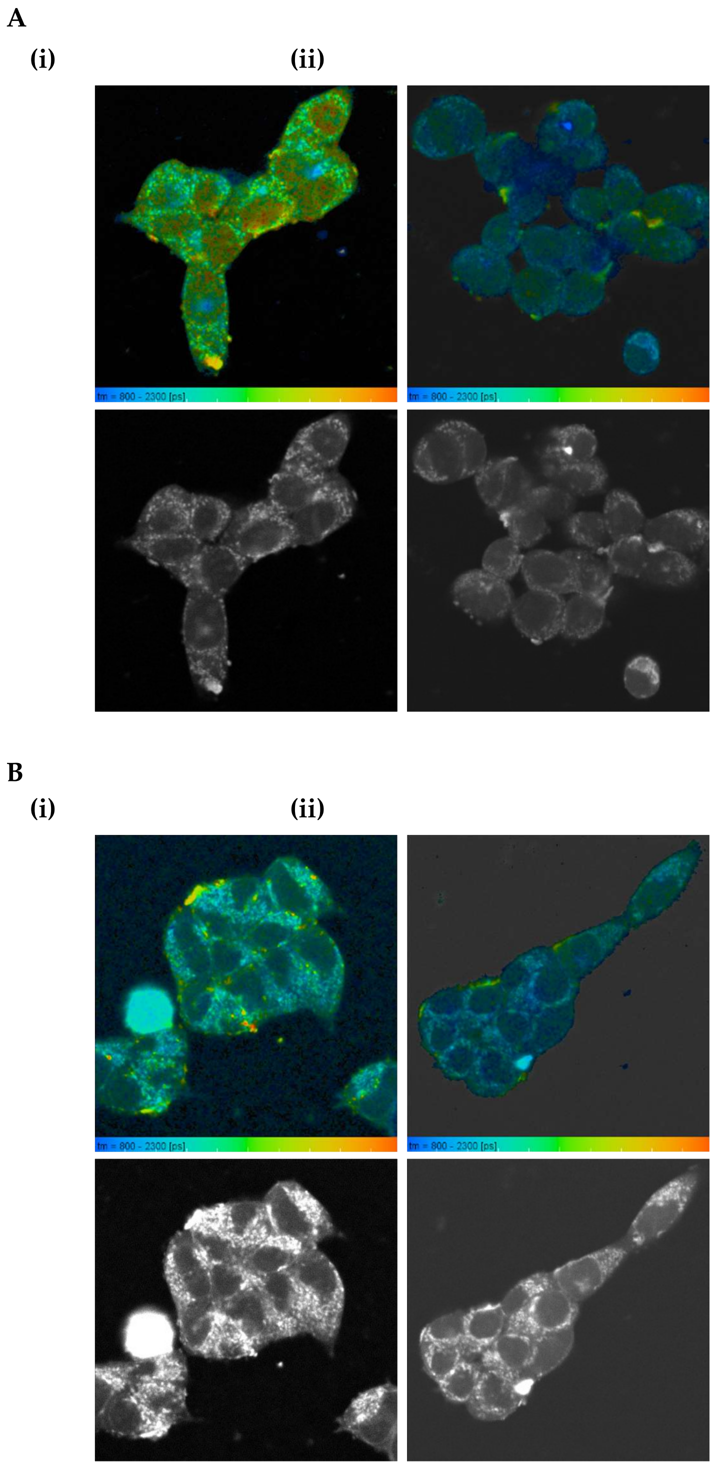

Figure S3. (i) Representative fluorescence lifetime (colored) and intensity (black & white) images of HEK293 cells incubated for 30 min with 50 μM of 9-Me-nHo and 9-Me-Ho (**A** and **B**, respectively), recorded under two-photon excitation at wavelength 840 nm, using the bp allvis emission filter. Column **(ii)** depicts representative fluorescence images of wt HEK293 cells in the absence of βC. The mean values of average fluorescence lifetime (μ / ns) are: **(A.i)** 1.57, **(A.ii)** 0.94, **(B.i)** 1.20 and **(B.ii)** 1.00.

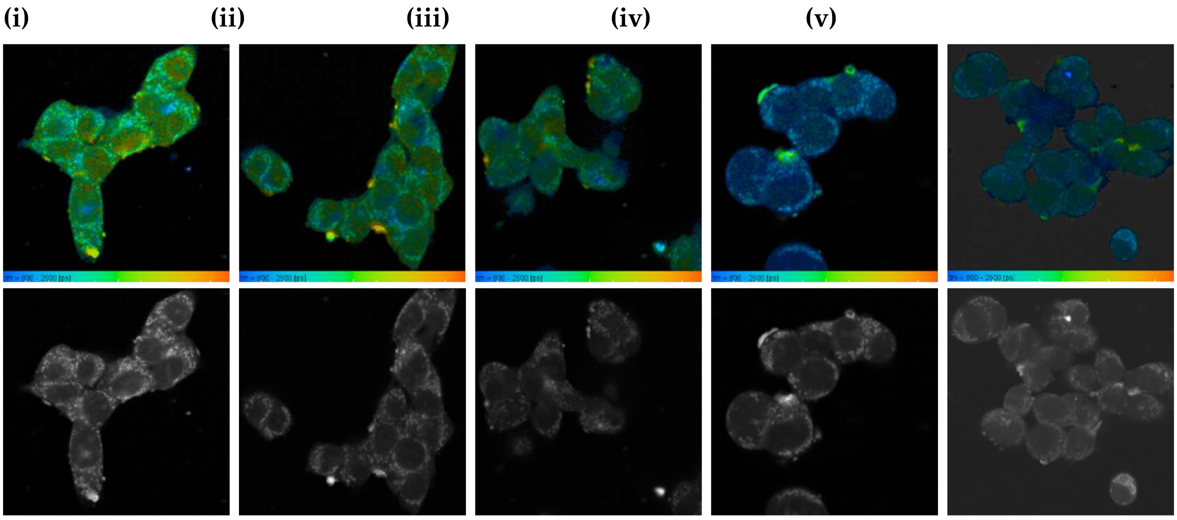

Figure S4. Representative fluorescence lifetime (colored) and intensity (black & white) images of HEK293 cells recorded under two-photon excitation at wavelength 840 nm, using the bp allvis emission filter. Columns from **(i)** to **(iv)** depict fluorescence images of HEK293 cells incubated for 30 min with 9-Me-nHo (50 μM) and then washed with fresh media, recorded every 5 minutes, respectively. The mean value of average fluorescence lifetime (μ / ns) changes from **(i)** 1.57, **(ii)** 1.52, **(iii)** 1.37 and **(iv)** 0.95. Column **(v)** depicts a representative fluorescence image of wt HEK293 cells in the absence of 9-Me-nHo (autofluorescence, μ = 0.89).

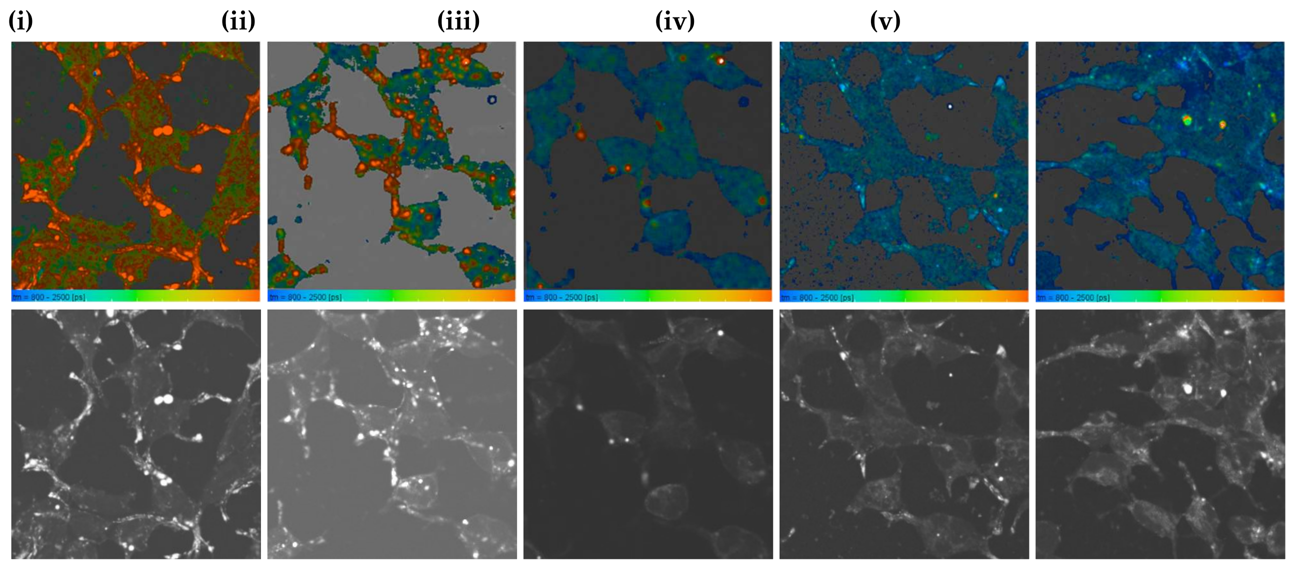

Figure S5. Representative fluorescence lifetime (colored) and intensity (black & white) images of HEK293 cells recorded under two-photon excitation at wavelength 840 nm, using the bp allvis emission filter. Column **(i)** depicts fluorescence images of HEK293 cells incubated for 30 min with 9-Me-Ho (50 μM) recorded in the presence of the βC (50 μM) in the external medium (EM). Columns from **(ii)** to **(iv)** depict fluorescence images recorded at 5, 12 and 45 min after washing the cells with fresh media. The mean value of average fluorescence lifetime (μ / ns) changes from **(i)** 2.85, **(ii)** 2.29, **(iii)** 1.16 and **(iv)** 1.05. Column **(v)** depicts a representative fluorescence image of wt HEK293 cells in the absence of 9-Me-Ho (autofluorescence, μ = 0.98).
